# Families Facing Pandemic Modifications of Everyday Life: A Mixed Study on Mothers’ and Children’s Emotional Reactions and Regulation Strategies

**DOI:** 10.3390/children10101627

**Published:** 2023-09-29

**Authors:** Giulia Carlotta Guerra, Odette Nardozza, Alessandra Frigerio, Maria Concetta Garito, Silvia Ponzetti, Ilenia Passaquindici, Mirco Fasolo, Maria Spinelli, Francesca Lionetti

**Affiliations:** 1 Department of Neuroscience, Imaging and Clinical Sciences, University G. D’Annunzio Chieti-Pescara, 66100 Chieti, Italy; giulia.guerra@unich.it (G.C.G.); odettenardozza@gmail.com (O.N.); mc.garito@libero.it (M.C.G.); s.ponzetti@unich.it (S.P.); passaquindici.ilenia@unich.it (I.P.); mirco.fasolo@unich.it (M.F.); francesca.lionetti@unich.it (F.L.); 2 Department of Psychology, Sigmund Freud University Milano, 20143 Milan, Italy; a.frigerio@milano-sfu.it

**Keywords:** COVID-19, mixed method, family wellbeing, coping strategies, mother, children, adolescents

## Abstract

The COVID-19 pandemic caused many enduring changes in the everyday life of families, with negative effects on parents’ and children’s wellbeing. However, there is a lack of studies in the literature exploring the emotional reactions and coping strategies of both mothers and children of different ages. Furthermore, most studies used only self-reports. This study aimed to identify the emotions and coping strategies of children, adolescents and their mothers and to verify the association between maternal and child wellbeing. A mixed-method design using interviews and questionnaires was applied to collect information on wellbeing (emotional reactions, behavioral/emotional problems) and coping strategies of both mothers (*n* = 65; M age = 42.17; SD = 4.40; M age = 41.63; SD = 4.48), and their children (*n* = 35, 8–10 year; *n* = 30, 11–13 year) during the second wave of the pandemic (December 2020). No differences between the groups emerged concerning the emotional reactions reported. In contrast, mothers and children of different ages reported different self-regulation and other-regulation strategies. Moreover, maternal strategies had different effects on children’s wellbeing. The integration of qualitative and quantitative results was informative to understand how families adapted to the radical changes of everyday life related to the pandemic. The implications for developing interventions in such similar stressful situations to promote family wellbeing are discussed.

## 1. Introduction

Between the end of 2019 and the beginning of 2020, the COVID-19 pandemic began, affecting the life of people all around the world with effects that are still present. On March 9th, the Italian population was forced to stay indoors to avoid the spread of this new virus. Families were forced to spend time in the same physical space for three months. Smart working and distance learning were introduced, school closures were compulsory and meetings in public spaces were forbidden. Some of these limitations and the several modifications of everyday social life aimed to contain infections such as the use of masks, social distancing, distance learning etc., lasted for months after the first lockdown.

This situation negatively affected families’ routines and behaviors [1,2] with negative effects on parents’, children’s and adolescents’ general mental health [2,3,4,5,6].

Previous studies conducted after potentially traumatic disasters evidenced that the potential short and long negative effects of stressful circumstances on children’s mental health is mediated by how the family handled the stress. For example, the quality of the caregiver supportive verbal communications to the child (i.e., caregiver’s willingness to discuss problems in general, providing encouragement, emotional expression) was associated with children’s positive coping strategies after the 2010 Chilean earthquake [7], with less negative psychological outcomes 11 years after the Chernobyl nuclear disaster [8], and with less anxiety and distress in adolescents [8,9]. On the other hand, when the distressed caregivers were unable to support their children’s ability to express and process painful emotions related to environmental trauma, the effect on the child was detrimental [7].

During and right after the beginning of COVID-19, many scholars aimed to explore the effects of everyday life modifications on children and parents’ wellbeing in several different countries, i.e., [10,11,12]. However, most of the studies investigated children’s wellbeing using parent reports, rarely explored both parents’ and children’s perspectives and focused on the first months of COVID-19 leaving the long-term effects unexplored. Moreover, most of them included wide age ranges failing to capture the differences in the emotional reactions between children and adolescents [10,13].

To contribute to this field of studies, the present research, using both quantitative and qualitative data, explored the emotional reactions and coping strategies of children, adolescents, and their mothers nine months after the beginning of COVID-19, in December 2020. This moment in time was very critical; even though the first lockdown had ended, the frequency of infections was still high. Children were requested to go to school following many restrictions (i.e., wearing masks, keeping social distance, reducing interactions, and studying via distance learning after contact with an infective person) and social lives were strongly modified. An in-depth exploration of children and parents’ emotional reactions might help in understanding the impact of such strong modifications of family life on parents and children of different ages. The findings will inform the design of clinical and psychosocial interventions directed at families during this and similar situations.

### 1.1. The Impact of COVID-19 Pandemic on Families’ Emotional Wellbeing

#### 1.1.1. Children’s and Adolescents’ Emotional Reactions

The majority of studies exploring children’s emotional reactions to COVID-19 related to modifications of their everyday life were conducted during the first lockdown (March–April 2020); only a few of them directly collected children’s opinions using mainly parent reports.

When interviewed about their wellbeing, children reported mixed emotions linked to the different impacts of the situation on their life. Missing their grandparents, playing with their friends, being able to go outside and having missed milestone events, such as birthday celebrations or graduations, made children interviewed in China, Spain and Australia feel bored, lonely, and sad [10,14,15,16]. On the other hand, the forced cohabitation made them feel happy, relaxed to be in their own home, helped the development of close family bonds and, at the same time, made them feel angry and worried about their family condition and nervous and agitated about the situation [10,13,14,15]. Moreover, children had low motivation and dissatisfaction about learning online, but they enjoyed some aspects of not going to school [10]. Not having appointments or class activities reduced the children’s stress levels and increased their free time for pleasure activities or for mastering new skills (e.g., yoga, video games, painting) (as reported in studies conducted in China and Austria) [10,13].

It is well known that the pandemic impacted lives for many months worldwide, with implications that are still present even if the emergency is almost over. However, only a few studies explored the long-lasting effects of COVID-19 modifications on everyday life after the first lockdown. Moreover, most of the studies considered a wide range of ages, failing to explore the specificities of children and adolescents’ reactions. The results of a qualitative study conducted in Austria a few months after the lockdown showed how children’s (from kindergarten age to 18 years old) emotional reactions varied according to age and were quite different from what was reported in studies conducted during the lockdown [13]. Specifically, younger children showed COVID-19 preoccupations by incorporating them into their games or recommending their parents to respect containment measures. Older children, instead, suffered from the lack of privacy and the reduction of social activities outside the family household (i.e., school, peer groups, recreational activities, visits to grandparents) [13]. Regardless of their age, children’s emotions changed from fear and anxiety, expressed during the first lockdown, to frustration and aggression shown a few months later [13].

To sum up, the lockdown had a mixed impact on children’s wellbeing. Scholars also reported the presence of positive reactions related to some aspects of modifications of life. An exploration of both positive and negative emotional reactions is necessary to fully understand the complexity of the experience. Moreover, emotional reactions could differ among ages with children and adolescents, experiencing two crucial developmental periods, showing different emotional reactions and different strategies used to cope with those emotions. While children might rely more on the family for emotional support and to share time and pleasure, adolescents are focused on relations with their peers, which often conflicts with parents. A crucial limit of the literature is the only focus on the lockdown experience; this was very useful to understand the immediate effects of the pandemic. However, understanding the long-lasting effects of the modifications of everyday life still present months after the beginning of the emergency and how that could be replicated if similar situations happen again is now necessary.

#### 1.1.2. Parents’ Emotional Responses

The changes in everyday life caused by COVID-19 also had a negative impact on parents’ wellbeing. The major COVID-19 pandemic’s restrictions and the rules imposed by the government to prevent contagions made parents feel angry, frustrated, and stressed by daily inconveniences, especially related to school, work and family members (as reported in a study conducted in the United Stated) [10]. In particular, trying to balance work, parenting and schooling children from home was perceived as very stressful [6]. Having to choose between prioritizing work at home or parenting made them feel guilty and unable to fully attend either role. Parents from the United States reported struggling in managing sibling relationships and in giving children limited opportunities for socialization with peers [17]. As for the work, unemployment, job insecurity and layoffs made parents feel high levels of stress [17].

With the extension of the first lockdown, parents expressed concerns about the long-term impacts of the COVID-19 pandemic on their children’s lives, specifically their mental health, education, and opportunities [10]. In addition, with the increase of deaths and infections, concerns were emerging regarding the impact of COVID-19 on the health of elderly parents, family members or friends and parents who feared becoming sick or dying [10,18]. The closure of infant centers and schools made parents experience fear of not feeling able to take care of their children in the event of infection of both parents [10,17]. In general, both parents in Italy and in the United States reported the fear of being unable to control what was happening to their family life [17,19,20].

Because the family condition impacted that much on parents’ emotional reactions, we might expect to find differences between the parents of children’s and adolescents’ reactions. However, to the best of our knowledge only one study conducted in Austria explored the difference between the negative emotions of parents of children of different ages [13]. Parents of kindergarten-aged children were concerned that their children had few social learning opportunities with their peers and were not receiving adequate preparation for school entry, and, as the duration of the first lockdown increased, they feared that children might forget previously learned kindergarten routines and their friends [13]. In contrast, parents of elementary school-age children showed concerns about their children’s educational disadvantages related to home learning due to limited abilities to support them [13].

As for children, the COVID-19 pandemic had some positive effects on parents’ wellbeing. Among the reported positive aspects of forced cohabitation, one was the creation of stronger bonds with family members and the ability to spend more quality time with their children and partner. In addition, improvements in sibling relationships were observed that had not occurred when schools were open [10,15,17].

In conclusion, the COVID-19 pandemic had both positive and negative effects on parents’ emotional sphere. Their emotional reactions varied according to their offspring’s age; however, only few studies explored these differences. In addition, most studies were conducted during the first lockdown, leaving a gap in the knowledge of long-term effects of the COVID-19 pandemic on parental wellbeing.

### 1.2. Coping Strategies to Regulate COVID-19 Emotional Distress

#### 1.2.1. Children and Adolescents’ Coping Strategies

Coping strategies refer to the regulatory processes people used to manage their emotions and behaviors under stress [21]. They are multidimensional and have different functions that enable a person to adapt to the environment. Coping strategies include controlling one’s emotional state by acting on it, self-regulation, and strategies aimed at gaining control of one’s emotional state by acting externally, other-regulation [21]. The first category includes behaviors such as reading a book, meditation, playing sports and all the action a person can do to regulate her/his emotion by his/herself. The second category includes all the behaviors aimed at seeking the help of an external person such as talking with someone about the issue, sharing ideas and thoughts and sharing or asking for affection.

Studies reported that children of different ages from Spain and the United States used several coping strategies to regulate the emotional distress caused by the COVID-19 pandemic [22,23]. Pre-schoolers predominantly used behavioral strategies such as “scream or get angry” to deal with their negative emotions [23]. On the other hand, school-age children used more engaged-oriented strategies such as problem solving to take specific actions to solve the crisis, seeking instrumental social support to try to understand how COVID-19 happened [23]. The coping strategies of preadolescents turned out to be more varied and complex, both cognitively and behaviorally. Children between 10 and 12 years used cognitive strategies as wishful thinking in which they hope the COVID-19 crisis had never happened or they reminded themselves that the lockdown situation was not that bad. Concerning emotional strategies, they tried to calm themselves down, they shared with their parents how they felt about the COVID-19 situation and used humor to joke or tried to laugh about the situation [23].

Some scholars found that specific pre-COVID-19 frequently used coping strategies were associated with children’s emotional wellbeing during lockdown (as reported in studies conducted Spain, China, and the United States) [11,22,24,25]. For 8–13-year-old children, the frequent use of savoring before COVID-19, a process of attending, intensifying and prolonging positive memories, was associated with greater stable positive affects during the lockdown, while the frequent use of dampening, a strategy used to down-regulate positive emotions, predicted low positive affects. In contrast, more frequent pre-COVID-19 rumination predicted the increase of negative affects probably because this dysfunctional strategy consists of thinking often about the negative aspects of a situation [22].

While younger children’s emotions were still externally regulated by their parents, adolescents gradually learned to self-regulate their emotions, showing different resources than children to cope with the COVID-19 inconveniences and to make new meaning of experiences related to the lockdown (as reported in an Italian study) [26]. Appraisal, the process that determines if a situation is perceived as stressful, positive thinking, acceptance and emotional awareness were considered as protective factors for adolescent’s wellbeing. The use of these coping strategies before COVID-19 was associated with low stress during lockdown in Spain and China [11,25]. On the contrary, procrastination was considered as a risk factor because it is a short-term emotion regulation strategy, based on avoidance, that does not allow processing and accepting negative emotions [11]. Additionally, through direct access to electronic devices (e.g., smartphones and computers) Chinese and Spanish adolescents were able to maintain social contacts to cope with negative emotions that were caused by the inability to meet with relatives and friends [24,27,28].

#### 1.2.2. Parents’ Coping Strategies

Parents’ strategies to deal with the pandemic’s impact on their personal and family life consisted of the tendency to regulate their own emotions and to regulate their children’s emotions.

Spending time outdoors in their own backyard and accepting that the lockdown was not up to them helped to enjoy moments and to maintain their wellbeing (as reported in studies conducted China and United States) [17,29]. Other parents used avoidance strategies toward elements that were a source of stress for them. For example, they limited contact with friends or relatives with whom they did not have a good relationship with, they avoided arguments with family members, and they limited access to news about COVID-19. One of the most used other-regulation strategies was maintaining contact with friends and family online [17]. Having supportive and understanding colleagues helped them decrease the stress generated by managing work and home life [17].

Parents’ strategies to regulate children emotions impacted on children’s wellbeing. Negative parental coping strategies, such as rumination and communication difficulties, were associated with high perceived stress in both parents and children in the United States [30]. Moreover, during the first lockdown in China, the United States and Spain, parental involvement, measured as the focus of the parent on children’s emotional experience, was positively associated with children and adolescents’ emotion regulation skills and low negative reactions [12,25,29,31,32]. On the other hand, parents with high levels of stress interacted less with their children, negatively affecting the child’s emotional regulation skills [12].

One of the major strategies that parents used to protect children from the negative impact of the pandemic was structuring family life during the lockdown to maintain as much as possible the habits and schedules of everyday life. This was conducted with attention to promoting the independence of their children by involving them in the care of the house or by giving them responsibilities. For example, younger children set the table or helped prepare food, while older children assisted with groceries, helped parents to take care of younger siblings or helped them with schoolwork. This strategy was employed in Chinese families with children of all ages, but the routine of young children was structured primarily by the parents, while adolescents were actively included in its creation and supported in respecting it independently [13]. An additional strategy was to provide children with a feeling of reassurance and family cohesion through parental closeness, communication, and the scheduling of shared activities (as reported in studies conducted Spanish, China and United States) [11,13,33,34]. Cohesion was established with frequent physical and emotional closeness with the child, such as devoting more time for cuddling [35] and through the sharing of activities that allowed for positive memories and strengthening of family bonds (i.e., walking, board games, movie nights and cooking) [13].

To regulate negative emotions caused by children’s exposure to COVID-19 news, parents of children preferred to communicate information with their own words or to use a restrictive mediation style to avoid exposing them directly to false or negative COVID-19 news [13,26]. In contrast, co-viewing was the style mainly used by the parents of pre-teens. Both restrictive mediation and social co-viewing styles were negatively correlated with children’s emotional functioning, probably because these mediation strategies did not allow for the organized processing of negative COVID-19 news. Regardless of children’s age, an active communication style used by parents to talk about negative and positive emotions related to COVID-19 news was associated with both a child’s greater ability to regulate their emotions as it allowed them to process information on COVID-19 at an emotional and cognitive level [26], and with more emotional problems in children, probably because talking a lot about the effects of the COVID-19 pandemic could cause them continued worry and rumination (as reported in studies conducted in Italy and United States) [27].

Older children were also encouraged to use social media and electronic devices for home education and to maintain social contacts independently [13].

In summary, during the lockdown, parents used several coping strategies to manage their own distress and to manage their children’s emotions and behaviors. While the latter were more explored, little is known on the strategies used by parents to cope with their own distress. Studies conducted in several countries evidenced that children and adolescents who were exposed to familiar protective factors, such as emotional support from parents, a well-defined daily routine and good communication with friends and parents, were more resilient to pandemic stress. On the contrary, children and adolescents who lack familiar protective factors are more likely to develop anxiety and depression [36]. Moreover, the quality of parenting support seems to be affected by parent’s wellbeing [36]. For these reasons, the study of both parental emotional reactions, parenting strategies, and the exploration of the link with children’s wellbeing is necessary.

### 1.3. The Present Study

We conducted a mixed-method study using questionnaires and interviews directed at mothers and children of different ages nine months after the beginning of the first lockdown (December 2020). The use of this method filled a gap in the literature because current studies examined these issues using only questionnaires, mostly filled in online. The choice to use a mixed method was made to obtain more information to help better understand which emotional reactions were associated with COVID-19 difficulties and which emotion regulation strategies were applied by mothers, children, and adolescents. In addition, the use of this method fills a gap in reading, as current studies address these issues using only quantitative methods, predominantly with online parent report questionnaires. Moreover, our study is original because it addresses these issues by dividing subjects into age groups and considering both the mother’s and child’s perspectives. An additional limitation of the literature that we aimed to fill is that most studies were conducted during the first lockdown, allowing only emotional reactions to be captured in the early period of the pandemic. Our study was conducted in Italy, one of the first countries affected by the pandemic after China. Even if the literature is quite homogeneous in findings of children and parenting reactions to the pandemic in several countries with different cultural background, an exploration of the situation in Italy and a comparison with other countries’ findings would be an important contribution to the literature.

The main aim of the study was to explore the emotional reactions and emotional coping strategies used by children, adolescents, and their mothers with the following specific aims:

(1) To identify emotional reactions in children, adolescents, and their mothers.
(2) To identify the emotional coping strategies used to cope with the COVID-19 pandemic by children, adolescents, and their mothers.
(3) To identify the association between maternal wellbeing and emotional and behavioral problems in children and adolescents.

Based on these objectives, we hypothesize that (1) emotional reactions and (2) coping strategies would differ by ages in the two groups (mother–child; mother–adolescent), and that (3) the psychological wellbeing of mothers is associated with that of their children/adolescents.

## 2. Materials and Methods

### 2.1. Participants and Procedure

Sixty-five mothers with their children participated in the study. Subjects belonged to a previous project. The sample consisted of 35 children (8–10 years old), 30 adolescents (11–13 years old) and their mothers. The final sample consisted of 65 mothers divided into two groups based on the age of their children: 35 mothers of children (M age = 42.17 (4.40) and 30 mothers of adolescents (M age = 41.63 (4.48). In terms of maternal education, the sample was composed of 9.5% less than a high school degree, 61.9% high school degree and 28.6% master’s degree. All participants were Italian, all mothers were married and/or co-habiting with the father of the child. Each family had an average of two children.

Mothers were contacted by phone in December 2020 and those who agreed to participate received the links to the online survey for the mother and the link to the online survey for the child. Appointments for the interviews (one with the mother and one with the child) were scheduled.

Children and their mothers signed the written consent form and completed the online survey composed by a set of questionnaires. Telephone interviews with mothers and children were conducted independently a few days after the first contact.

### 2.2. Measures

#### 2.2.1. Family Data

Mothers answered a set of specific questions related to their family condition. The first three questions addressed to what extent (on a Likert scale from 1 = very little to 7 = very much) when the pandemic was over, she thought she would need financial support and psychological support for herself and her child (i.e., To what extent do you think you will need economic support after the pandemic is over?; To what extent do you think you will need psychological support for you?; To what extent do you think you will need psychological support for your children?). Mothers were also asked how often their child asked questions, read or talked about COVID-19 (on a Likert scale from 1 = never to 5 = most of the time).

#### 2.2.2. Mothers’ COVID-Contact Risk Index

An ad-hoc index was computed to evaluate the amount of contact the parent had with people directly affected by the virus, following the assumption that the greater the number of contacts, and the closer to the parent were the people, the greater the impact on psychological wellbeing would be. One point was given for each of the following if present: the parents tested positive for the virus, a familiar or close friend tested positive, a familiar/close friend was hospitalized, a familiar/close friend died. A half point was given if the parent knew a person (not familiar or close friend) who tested positive, was hospitalized or died.

#### 2.2.3. Mothers’ Worries Regarding COVID-19

An ad-hoc index was computed to evaluate mothers’ concerns about possible COVID-19 infection and the consequences on their own and others’ physical and mental health (i.e., During the past month how concerned were you: about being infected; about the possibility that your friends and family members might be infected; about the possibility that your physical health might be affected by COVID-19; about the possibility that your mental health might be affected by COVID-19?). The questionnaire included four items (on a Likert scale from 1 = very little to 5 = very much). To obtain the score of each scale, items are summed. Cronbach’s α in the current study was 0.86.

#### 2.2.4. Mother’s and Child’s COVID-19 Difficulties

Difficulties experienced by parents and children during the pandemic were investigated with a newly developed pool of 13 items. Parents and children were asked to indicate (on a Likert scale from 1 = very little to 7 = very much) how difficult they were perceiving, during the last week, dealing with several aspects related to the COVID-19 period (i.e., Thinking about yourself, how difficult did you find it to balance family and work?; Thinking about yourself, how difficult did you find it to read a book?). To obtain the score of each scale, items are summed. Cronbach’s α in the current study was 0.85.

#### 2.2.5. Mothers’ Psychological Wellbeing

Individual perceptions of maternal wellbeing were investigated using short form of the Depression Anxiety Stress Scales-21 (DASS-21) [37]. The questionnaire has 21 items and provides three sub-scales. Each sub-scale is composed of 7 items (on a Likert scale from 0 = did not apply to me at all to 4 = applied to me very much or most of the time) to measure the individual symptoms indicating stress (i.e., I found myself getting agitated), depression (i.e., I was unable to become enthusiastic about anything) and anxiety (i.e., I was aware of the action of my heart in the absence of physical exertion). To obtain the score of each scale, items are summed. Cronbach’s α in the current study were the following: α = 0.87 for Depression symptoms, α = 0.81 for Anxiety symptoms and α = 0.86 for Stress symptoms.

#### 2.2.6. Mothers’ Dyadic Parenting Stress

The perception of parenting stress in the parent–child interaction was investigated using the sub-scale of dysfunctional parent–child interactions of the Parenting-Stress Index (PSI-4) [38]. The scale investigated the extent of parents’ agreement or disagreement with statements describing the parent–child relationship as difficult to manage (on a Likert scale from 1 = very much to 5 = very little) (i.e., my son rarely does things for me that gratify me; when my son plays, he does not laugh or show that he enjoys himself often; my son does not smile as much as most children do.). To obtain the score of each scale, items are summed. Cronbach’s α in the current study was 0.87.

#### 2.2.7. Child’s Emotional and Behavioral Problems (Child Report and Maternal Report)

Behavioral and psychological problems in children were investigated using both mothers’ and children’s reports of the Italian version of the Strengths and Difficulties Questionnaire (SDQ) [39]. The current study focused specifically on the following subscales: emotional symptoms (i.e., “I am often unhappy, sad or in tears”), hyperactivity–inattention (i.e., “constantly on the move or uncomfortable”) and conduct problems (i.e., “Often quarrels with other children or purposely annoys them”). Each subscale is measured by 5 items (on a Likert scale from 1 = not true to 3 = very true). To obtain the total scores, items are summed (i.e., respectful of others’ feelings; complains frequently of headache, stomachache or nausea; constantly moving or uncomfortable). Cronbach’s α in the current study were as follows: α = 0.53 for emotional problems; α = 0.51 for conduct problems and α = 0.77 for hyperactivity-inattention.

#### 2.2.8. Children’s Activities

Children answered a set of specific questions related to assessing the possible changes on technology use during the pandemic. Children were asked how much time (on a Likert scale form 1 = never to 5 = 4 and more hours) they played video games throughout the day (i.e., How many hours a day did you spend playing video games in the past month). They were asked how many times a day they heard their friends on a video call (i.e., How many times a day do you hear your friends and girlfriends on video call?) (from 1 = never to 3 = two or more time). In addition, how many hours they watched TV was investigated (i.e., How many hours a day did you spend watching TV in the past month?) (on a Likert scale from 1 = I never watched TV to 4 = more than 3 h). Finally, they were asked how often they searched for information about COVID-19 (i.e., During the past month, how often did you happen to read or talk about COVID-19?) (from 1 = never to 5 = almost always). To obtain the score of each scale, items are summed. The Cronbach’s α for this scale was 0.49.

#### 2.2.9. Children’ Worries Regarding COVID-19

As for mothers, an ad-hoc index was computed to evaluate children and adolescents’ concerns about possible COVID-19 infection and the consequences on their own and others’ physical and mental health. The questionnaire included four items (on a Likert scale from 1 = never to 5 = very much): During the past month how concerned were you: about being infected; about the possibility that your friends and family members might be infected; about the possibility that your physical health might be affected by COVID-19; about the possibility that your mental health might be affected by COVID-19?). To obtain the score of each scale, items are summed. The Cronbach’s α for this scale was 0.75.

#### 2.2.10. Children’s COVID-19 Difficulties

Difficulties experienced by children and adolescents during the pandemic period were investigated with a newly developed pool of 8 items. Children and adolescents were asked to indicate (on a Likert scale from 1 = not difficult at all to 5 = very difficult) how difficult they were perceiving, during the last month, dealing with several aspects related to the COVID-19 period such as falling asleep, waking up in the morning, getting along with my parents, staying away from my friends, etc. To obtain the score of each scale, items are summed. The Cronbach’s α for this scale was 0.69.

#### 2.2.11. Mothers’ and Children’s Emotional Reactions and Coping Strategies Associated with COVID-19 Pandemic

To explore the emotional reactions and strategies used to cope with COVID-19-related difficulties, telephone interviews were conducted with mothers and their children. For the children, the questions concerned the difficulties they experienced during the COVID-19 period at school, at home and with friends. They were also asked what emotions they felt about these difficulties and what strategies they used to cope with them (see Appendix A). For mothers, on the other hand, the questions focused on emotional reactions and coping strategies by referring to both their own difficulties associated with COVID-19 and those experienced by their children. In addition, mothers were asked to report what parenting strategies they used to cope with their children’s emotional reactions (see Appendix B).

### 2.3. Analytic Plan

#### 2.3.1. Quantitative Analyses

Mothers’ and children’s scores at each questionnaire were computed. Descriptive analyses were performed to examine the distribution of answers to questions. Differences between groups were examined with a one-way ANOVA. Associations among variables were examined with Pearson’s correlations in the two groups.

#### 2.3.2. Qualitative Analyses

A theoretical thematic analysis was used to identify the dominant themes across the entire data set. The data analysis was conducted according to Braun and Clarke’s Thematic Analysis framework [35,40]. We adopted a theoretical, deductive or “top down” way of performing the analysis. This means that the themes identified are strongly related to the specific research questions and the coding process is driven by the particular interest of the study. In this sense, this form of thematic analysis is analyst-driven and provides a detailed analysis of specific aspects of the data, which were fit into a predefined coding frame based on the specific research questions and hypothesis. In the case of this article, we were explicitly interested in the impact of the COVID-19 pandemic and the related coping strategies adopted by mothers and their children; thus, we specifically focused on these features and aspects in coding the data.

The type of analysis adopted can also be described as semantic, as the data were organized and summarized to underline patterns in the explicit content of participants’ responses. We focused on the meanings clearly included in the interviewees’ words, providing an accurate description of the data, rather than an interpretation that goes beyond what participants said.

First, the interviews were fully transcribed by a research assistant. Then, we proceeded with the familiarization with the data, reading the participants’ answers several times. Three members of the research team identified codes by selecting and tagging parts of the text considered pertinent to understanding the reactions and the coping strategies adopted by mothers and children to front the pandemic. They produced a list of extracted codes that was then discussed within the entire research group to define macro-areas relevant to the research questions. This progressive organization in themes was carried out by combining, splitting or redefining codes in order to reach consensual clusters of concepts featuring the research areas of investigation. All the data were analyzed in their original language, Italian.

In reporting the results, we decided to present the final list of themes and codes with exemplificative quotes from the interviews and include all of them in tables. We specified with participants our availability of answering doubts, questions and on giving them information and feedback when requested on the study’s findings.

## 3. Results

### 3.1. Descriptives

The exploration of frequencies of answers to the living characteristics questions gave interesting information about the two groups. In total, 44% of children declared spending 2 h or more per day playing videogames versus 23% of adolescents. Concerning video-calling friends, 47% of children and 47% of adolescents declared doing it at least 2 or more times a day. More than 50% of children and 30% of adolescents watch TV for more than 2–3 h a day. A total of 64% of children and 66% of adolescents reported reading and talking about COVID-19 often and always during the last month. Mothers of children and adolescents declare on average to need low economic (M children = 2.61, SD = 1.78; M adolescents = 2.59, SD = 1.93) and psychological (M children = 2.61, SD = 1.79; M adolescents = 2.38, SD = 1.42) support for themselves and psychological support (M children = 2.55, SD = 1.65; M adolescents = 2.48, SD = 1.57) for their children.

### 3.2. Differences between the Groups

One-way ANOVA results showed that mothers of adolescents reported higher parenting stress and less worries about COVID-19 than the mothers of children (see Table 1). No significant differences emerged between children and adolescents reporting emotional and behavioral problems.

#### 3.2.1. Associations among Maternal and Child-Reported Behavioral and Emotional Problems

Pearson’s correlations among maternal-reported and child-reported behavioral and emotional problems assessed with the SDQ were very high across all scales (mean r = 0.48) (Table 2). For this reason, we decided to keep only the SDQ data as directly reported by children and adolescents for further analyses.

#### 3.2.2. Associations among Maternal Wellbeing and Children Behavioral and Emotional Problems

Pearson’s correlations among maternal wellbeing (PSI—Parenting stress during the interaction, DASS—depression, anxiety and stress symptoms) and children’s emotional and behavioral problems (SDQ) evidenced significant associations in the two groups (see Table 3). Mothers of children with high parenting stress had children with more behavioral problems, while mothers of adolescents with higher parenting stress have children with higher hyperactivity and attention problems. Hyperactivity and attentional problems as well as emotional problems in adolescents were also associated with high depressive, anxiety and stress symptoms in mothers; this association was not present in the children group. High behavioral problems were associated with maternal high depression and stress in the group of children and with high anxiety and stress in the group of adolescents.

#### 3.2.3. Associations among COVID-19-Related Variables and Mothers and Children’s Wellbeing

Associations among COVID-19-related variables and wellbeing are presented in Table 4. A high frequency of searching information about COVID-19 and high level of worries about the pandemic were associated with higher emotional problems only in adolescents. High fatigue to adjust to COVID-19 modifications of everyday life was associated with high emotional and behavioral problems in the two groups and with hyperactivity/attention problems only in the children group.

A higher number of difficulties in adjusting to COVID-19 modifications of everyday life was associated with mothers’ high anxiety and stress only in the group of children. In both groups, more preoccupations about COVID-19 were associated with high maternal anxiety and stress, and with high depression only in the children group.

### 3.3. Qualitative Findings

#### 3.3.1. Mothers

The emergency situation associated to the world pandemic significantly impacted on the emotional, physical and cognitive levels of functioning of the participant mothers. Themes that emerged are described on Table 5.

The qualitative analysis, consistent with the quantitative results, showed that the themes that emerged from the interviews of mothers of children and mothers of adolescents were similar (see Table 5). Indeed, both groups reported experiencing similar emotional, physical, and cognitive reactions.

The main emotional reactions to the COVID-19 event among the mothers can be classified in the following categories: fear of being infected by and infecting other people; sense of vulnerability and powerlessness; feeling of irritability/agitation/frustration/anger; apathy and sadness; anxiety, worry and uncertainty; feeling of loneliness and inadequacy/insecurity.

Concerning the physical discomfort, COVID-19 impacted all mothers mainly in terms of sleep problems (i.e., insomnia, difficulties in sleep regularity and frequency of nightmares) and feelings of tiredness. Mothers of children also reported an impact in terms of psychosomatic reactions (i.e., increase in body weight, digestive problems, weakness and muscle rigidity, variations in menstrual cycle).

Regarding the cognitive impact of the situation, all mothers reported difficulties in concentration and lack of attention. While mothers of children described these difficulties in terms of feelings of confusion, mothers of adolescents experienced it as an overthinking tendency.

The coping strategies could be classified as two different types: self-regulation strategies and other-regulation strategies. Examining the maternal ability of managing the situation is particularly relevant because, as evidenced by the quantitative data, mothers’ levels of COVID-19-related worries are positively associated with the level of the offspring’s emotional difficulties. For both samples, self-regulation strategies included rationalization of emotions and thoughts in order to develop acceptance and pro-activity to front what is experienced as inevitable. Besides that, participants found comfort in practicing hobbies and sports, to keep their creativity alive, take care of their body–mind system and distract themselves from the problematic situation. In this respect, mothers of adolescents were more generic in reporting the ways they found to keep themselves busy, referring to these activities mainly as a way to avoid thinking at the situation. Mothers of children, instead, detailed the activities more in depth, referring to specific hobbies, sports and personal interests they dedicated time to. They also sought information and read up on the continuously evolving situation to better understand it.

Instead, mothers of teenagers adopted a self-isolation strategy—that was, being able to take some time alone to lessen the emotional overload—which instead was not mentioned by mothers of children, probably because they were not allowed to do so, given the age of their offspring. This difference seems to align with quantitative results that show that mothers of children had more worries related to COVID-19 and its impact than mothers of adolescents, who were probably less worried also due to the possibility of adopting temporary self-isolation as an adaptative regulation strategy.

Mothers who adopted other-regulation strategies specifically referred to a way to regulate their emotions based on interpersonal dialogue and searching for other people’s support. This covered both deep exchanges with relatives—who are part of, or live close to, the family nucleus—and more sporadic interactions with friends, neighbors, and acquaintances. As expected, both groups of mothers mainly relied on other members of the family nucleus, them being the only people with whom it was possible having face-to-face interactions during the pandemic. Concerning the ways mothers of both groups managed their children’s challenges of the emergency situation, we found several emotional and behavioral parenting strategies.

The emotional strategies first included engaging in an open and transparent dialogue with their children, which embraced: encouraging self-narration and disclosure about their feelings as well as explanation and clarification of what the situation is like and what they could expect in future. Mothers of teenagers experienced more difficulties in engaging in an open dialogue, so they tended to adopt a more directive style, giving concrete advice on how to manage time. This result supports and enlightens the quantitative analysis showing that mothers of adolescents are characterized by a higher level of parenting stress, but not a higher level of generic stress.

Second, mothers tried to appease and reassure, adopting a downplaying attitude to underline to children the transitory character of the situation and to adolescents its shared nature (that is, everybody was experiencing the same situation).

Other parenting strategies embraced pushing the children to engage themselves in daily activities and keeping their routines, as well as playing and sharing practical/intellectual activities together.

All the mothers tended to be more tolerant and indulgent toward the use of technological devices such as mobile phones and tablets, applying a less invasive attitude in order to give children and teens a space free from control.

Especially with children, mothers tried to take advantage of the enforced physical presence within the domestic environment and the related constrained proximity, to develop closer emotional intimacy and connection. On the contrary, mothers of adolescents tended to push them into seeing friends, when possible, to prevent the risk of social isolation.

#### 3.3.2. Children and Adolescents

Children and adolescents reported a number of emotional reactions as shown in Table 6.

First is the fear of contagion for oneself and significant others, which was more common among adolescents, who thought more at the possibility to ‘bring COVID-19 home’ and infecting the elderly family.

Second is worries and anxiety, which for children denoted a general sense of danger and referred to a number of scenarios: the possibility of losing other people, such as grandparents; school mates not respecting the COVID-19 rules; the preoccupation of unintentionally not respecting all the social limitations imposed by the pandemic (such as the use of masks); the difficulty in seeing the end of the pandemic, and thus the restoration of usual relationships, that end up in the worry of a never-ending story. For teenagers, worries and anxiety were specifically linked to the school context and to a general feeling of wasting time, and the uncertainty about the situation tended to end up in a self-closing mood.

Third is sadness and depressive mood, manifested through frequent and “unexplained” crying and associated to a general feeling of isolation, disconnection and distance from other people. In particular, both groups reported sadness due to: the great number of dead people; lack of affective interactions with both peers and the elderly; the impossibility to greet friends in cases of the transition from one school cycle to another; the nostalgia for a past era of freedom contrasted with a present and a prospective future full of restrictions.

Fourth is the feeling of boredom and lack of fun moments. Children especially reported that they struggled in finding what to do within the house walls, so they tended to spend a lot of time asking themselves what to do, while doing nothing. Adolescents especially associated boredom to a sense of decrease in creativity and motivation and to feelings of emptiness and loneliness.

Concerning the coping mechanisms adopted by children and adolescents, self-regulation strategies for both groups relied on hobbies and diversions, such as playing, reading, listening to music, playing music instruments, drawing, watching movies, TV, video games and, in a limited number of cases, reorganizing personal spaces. To deal with negative emotions, some children attempted to avoid thinking about the situation and adopted an apparently indifferent stance, while other children relied on positive thinking, i.e., recalling beautiful moments of the past spent with significant people and friends. Adolescents tended to keep emotions and thoughts for themselves, avoiding talking and sharing feelings with others.

Regarding the external strategies of regulation, children mainly count on the family context. For example, they tend to help parents in daily accomplishments and engage in activities with siblings. They sometimes directed a clear request for help, asking the other members of the family to spend time with them. Adolescents relied more on friends, with whom they interacted via video/phone calls and chatting, but also mentioned their parents as a source of support. Indeed, quantitative analysis showed that, among the teenagers, those with higher level of emotional difficulties tend to rely on parents especially for information seeking about COVID-19. Another form of external regulation mentioned by both the samples is represented by engaging in interactions with pets.

## 4. Discussion

The present study aimed to verify, during the period of the second lockdown, the emotional reactions and coping strategies used by children, adolescents and their mothers, and their associations. The mixed research design of the study, through the administration of self-reports and interviews, provided insight both on the specific strategies and emotional reactions of children and their mothers and into how the way mothers dealt with stressful situations related to the COVID-19 pandemic affected the wellbeing of children and adolescents. Furthermore, the division of the children’s sample by age helped in the identification of different patterns across groups.

In relation to the first hypothesis, no relevant differences between children and adolescents and between mothers of children and mothers of adolescents emerged in the reported emotional reactions. Regarding the emotional reactions of children and adolescents, the interviews revealed differences in the causes associated with the emotion (e.g., for the children, anxiety was associated with losing loved ones, whereas in adolescents with distance learning). The same emotions have been reported in previous studies, but none of these explored differences based on the age of the subjects [10,26,41]. In our case, the quantitative analysis allowed further differences to be identified. Adolescents tend to seek an explanation for the changes and limitations caused by COVID-19 as a reaction to the anxiety and worries experienced.

Consistent with the literature, mothers in both groups, during the second lockdown, reported similar reactions [10,17]. To the levels of anxiety and depression evidenced by the questionnaires corresponded similar emotional, psychosomatic and cognitive reactions. The use of a quantitative methodology alongside a qualitative methodology made it possible to identify differences between the two groups of mothers specifically concerning aspects more related to the parenting experience. To the intensified parenting stress reported by mothers of adolescents corresponded a greater difficulty in finding appropriate ways to cope with the situation such as dialoguing with the kid instead of using a directive and more authoritarian parenting style. We might suppose that during this moment of crisis characterized by social isolation and the necessity to respect many limits to everyday life, parenting an adolescent was a more challenging experience [10].

In relation to the second hypothesis, the results helped to fill the gap in the literature on the differences between self-regulation and other-regulation strategies used to cope with the changes related to the stressful situation. Children used more cognitive avoidance to cope with negative emotions, whereas adolescents engaged more in extra-familiar activities and hobbies as self-regulation strategies. Maybe, adolescents who possess more cognitive strategies to understand the stressful event were able to choose activities that allow them to self-regulate. As other-regulation strategies, children sought help from family members, whereas adolescents relied on friends, using social media [27,28]. This is consistent with specificities of the ages, with adolescents giving more importance to the peer group. In addition to previous findings, caring for and spending time with one’s pets emerged as an other-regulatory strategy in both children and adolescents.

The interviews reveled that in both groups, mothers used self-regulatory strategies, such as engaging in hobbies and seeking information about COVID-19 [17]. However, teen’s moms used self-isolation as a coping strategy. Having older children allowed them to take more time for themselves, which may explain why teens’ mothers reported lower concerns about COVID-19. Among the other-regulation strategies, mothers relied on their partner and family members. For the strategies used by mothers in managing their children, differences between groups emerged from the interviews. For example, consistent with previous findings, children’s mothers used more physical contact and closeness to establish a greater emotional connection compared to mothers of adolescents [13]. Furthermore, our results underlined how adolescent’s mothers found difficulties in establishing an open dialogue with their children about the emotions they felt, thus assuming a directive or permissive attitude to manage the situation during the second wave of the pandemic. On the behavioral level, both groups reported to share moments together with their kids and urged them to maintain the routine of the pre-pandemic period [13]. Mothers also reported a greater than usual tolerance in allowing their children to use technology and in pushing adolescents to meet their friends, when possible, to avoid social isolation. This represents a new element specific to the second wave of COVID-19 not found in previous studies conducted during the first phase of the emergency [31].

Finally, in relation to the third hypothesis, the quantitative results evidenced a significant association between maternal and child wellbeing. This contributed to understanding how the way the mother handled a stressful event influenced the child’s psychological wellbeing [27]. In the adolescent group, emotional and behavioral problems were positively associated with maternal stress, anxiety and depression. It is known that adolescence is often characterized by mother–child communication problems [27], and the way adolescents’ mothers reported handling the stressful event impacted more on this issue. Interestingly, in the group of children high maternal symptoms of anxiety and stress were associated with children’s difficulties in adapting to changes in daily life. Children and mothers with non-functional strategies had more difficulties in coping with the stressful event, and greater emotional and behavioral problems. Moreover, mothers with low psychological wellbeing might have problems in communicating with their children and in being supportive to their emotion regulation [12,30]. Finally, both quantitative and qualitative results show that how the parents manage the pandemic influences the way in which children and adolescents in turn manage it.

Despite the important results reported, the present study presents some limitations. First, the small sample, and the homogenous cultural context where the study was conducted, limit the generalizability of findings. Second, the cross-sectional design limits the possibility to explore causations. Third, we did not explore the emotional reactions and coping strategies of other family members, such as fathers. Finally, even if we know that the economic level of our participants was middle-high, we do not have detailed information about the socio-economic condition of the families. Future longitudinal studies on the topic investigating the long-term effects of the pandemic on the psycho-emotional wellbeing of both children and adolescents, in larger samples and including the full family perspectives are needed.

Despite these limits, the present study gives a picture of how mothers and their children coped with the challenges related to COVID-19′s modification of everyday life. This further underlines the need to use multi-informant exploration of phenomena. Moreover, our results are consistent with the literature concerning other cultural contexts, and consequently could not be considered culturally specific. This is important, because even if the different countries reacted differently to the pandemic applying different restrictions, the impact on families was similar and mainly related to the restrictions of social life such as the closing of schools and the absence of opportunities to meet with peers and relatives. The use of a mixed methodology evidenced how the solo use of questionnaires or interviews, as well as only the use of one respondent, limit the complexity of findings. Both scholars and clinicians should include in their exploration of the phenomena information from several perspectives, and their integration in order to increase their understanding.

Our findings contribute to capturing important information about how the families of children and adolescents may react when faced with stressful events that may involve the entire family system (such as natural cataclysms, wars, bereavements, etc.). In this way, intervention as well as preventive projects should be developed considering the specific family needs in relation to the age of the children.

In conclusion, it is recommended to intervene on the parent–child interaction to promote family wellbeing. In general, interventions should be based on promoting the maintenance of daily routines (i.e., for children, establishing routines to create expectations about the continuation of the day, and for adolescents, create routines to involve them in activities of daily living or make them participate in daily life events), emotional closeness of parents and fostering an open dialogue to enable children and adolescents to understand and emotionally manage stressful events. In addition, it would be useful to promote parental support projects to help them decrease the stress caused by the everyday family routine modifications to foster more adaptive parenting, a fundamental variable to prevent children and adolescents’ emotional and behavioral problems.

## Figures and Tables

**Table 1:** Differences between the groups.

	Children		Adolescents		*F* (1, 59)	*p*	*µ_p_*
	M	SD	M	SD			
Parenting Stress	21.63	7.34	25.59	8.25	3.94	0.05	0.06
DASS Depression	13.03	5.01	13.69	4.38	0.30	0.59	0.01
DASS Anxiety	11.53	3.98	11.66	4.47	0.01	0.91	<0.01
DASS Stress	14.63	4.53	15.24	4.37	0.29	0.59	<0.01
Economic Support	2.61	1.78	2.59	1.94	0.00	0.96	<0.01
Psychological Support Mother	2.61	1.78	2.38	1.42	0.31	0.58	0.01
Psychological Support Child	2.55	1.65	2.48	1.57	0.02	0.88	<0.01
COVID-19 Difficulties Mother	44.41	15.08	39.14	14.76	1.89	0.17	0.03
Worries Mother	11.88	3.56	9.62	3.27	6.59	0.01	0.10
SDQ Hyperactivity/Attention	9.12	1.85	8.77	1.43	0.70	0.40	0.01
SDQ Emotional Symptoms	8.24	2.24	8.63	2.46	0.46	0.50	0.01
SDQ Behavioral Problems	8.09	2.23	8.17	1.76	0.02	0.88	<0.01
COVID-19 Difficulties Child	22.97	5.66	21.40	4.92	1.38	0.24	0.02
Worries Child	10.79	3.97	10.10	3.68	0.52	0.47	0.01

Abbreviations: DASS (Depression Anxiety Stress Scales); SDQ (Strengths and Difficulties Questionnaire).

**Table 2:** Pearson’s Correlations among Maternal and Children Reported Behavioral and Emotional Problems.

	SDQ Hyperactivity/Attention ^1^	SDQ Emotional Symptoms ^1^	SDQ Behavioral Problems ^1^	SDQ Hyperactivity/Attention ^2^	SDQ Emotional Symptoms ^2^	SDQ Behavioral Problems ^2^
SDQ Hyperactivity/Attention ^1^	-	0.337	0.649 **	0.485 **	0.440 *	0.296
SDQ emotional symptoms ^1^	0.451 **	1	0.266	0.261	0.476 **	0.138
SDQ behavioral problems ^1^	0.511 **	0.552 **	1	0.576 **	0.280	0.554 **
SDQ Hyperactivity/Attention ^2^	0.475 **	0.220	0.379 *	1	0.250	0.358
SDQ emotional symptoms ^2^	0.448 *	0.510 **	0.223	0.488 **	1	0.532 **
SDQ behavioral problems ^2^	0.489 **	0.614 **	0.483 **	0.480 **	0.721 **	1

Note: ** *p* > 0.01, * *p* > 0.05; ^1^ mother report; ^2^ child/adolescent report.

**Table 3:** Pearson’s correlations among Maternal Wellbeing and Children Behavioral and Emotional Problems.

		PSI	DASS Depression	DASS Anxiety	DASS Stress
Children	SDQ Hyperactivity/Attention	0.34	0.29	0.27	0.19
	SDQ Emotional Symptoms	0.35	0.20	0.17	0.17
	SDQ Behavioral Problems	0.58 **	0.43 *	0.26	0.38 *
Adolescents	SDQ Hyperactivity/Attention	0.37 *	0.68 **	0.65 **	0.63 **
	SDQ Emotional Symptoms	0.09	0.35	0.51 **	0.45 *
	SDQ Behavioral Problems	0.18	0.30	0.51 **	0.45 *

Note: ** *p* > 0.01, * *p* > 0.05. Abbreviations: PSI (Parent Stress Index); DASS (Depression Anxiety Stress Scales); SDQ (Strengths and Difficulties Questionnaire).

**Table 4:** Pearson’s correlations among COVID-19-Related Variables and Mothers and Children Wellbeing.

		PSI	DASS Depression	DASS Anxiety	DASS Stress	SDQ Hyperactivity/Attention	SDQ Emotional Symptoms	SDQ Behavioral Problems
Children	Search for information	0.07	−0.17	0.05	−0.12	0.21	0.29	0.18
	Worries Child	−0.14	0.19	0.16	0.25	−0.10	0.20	0.24
	COVID-19 Difficulties Child	0.11	0.09	0.22	0.16	0.42 *	0.42 *	0.52 **
	COVID-19 Difficulties Mother	0.26	0.26	0.42 *	0.45 **	−0.11	0.18	0.15
	Worries Mother	0.10	0.54 **	0.60 **	0.53 **	0.02	0.08	0.03
Adolescents	Search for information	0.11	0.09	0.29	0.48 *	0.00	0.43 *	0.06
	Worries Child	−0.08	0.25	0.34	0.26	0.15	0.47 **	0.11
	COVID-19 Difficulties Child	0.33	0.07	0.11	0.26	0.23	0.38 *	0.37 *
	COVID-19 Difficulties Mother	0.28	−0.29	−0.05	0.17	−0.21	0.25	−0.03
	Worries Mother	0.10	0.33	0.56 **	0.48 **	0.32	0.47 **	0.30

Note. ** *p* > 0.01, * *p* > 0.05. Abbreviations: PSI (Parent Stress Index); DASS (Depression Anxiety Stress Scales); SDQ (Strengths and Difficulties Questionnaire).

**Table 5:** Themes and Sub-themes with Quotes from Mother’s Interviews.

Mothers of Children	Mothers of Adolescents
Emotional reactions	
Fear of infection ^1^	Fear of infection
Sense of vulnerability and powerlessness ^2^	Sense of vulnerability and powerlessness ^4^
Feeling of irritability/agitation/frustration/anger	Feeling of irritability/agitation/frustration/anger ^5^
Apathy and sadness	Apathy and sadness
Anxiety, worry and uncertainty ^3^	Anxiety, worry and uncertainty ^6^
Feelings of loneliness and inadequacy/insecurity	Feelings of loneliness and inadequacy/insecurity
^1^ “*I was afraid of getting sick, afraid of my father getting sick…of not being able to protect the people around me, this was the scariest thing”* ^2^ “*There was this whole atmosphere of anxiety, of fear. Because you didn’t know what you’re up against; in the evening I felt sadness, uncertainty, fear…”* ^3^ *“Certainly more fragile, because I no longer had the situation in my hands, and it no longer depended on you”*	^4^ “*I happened to feel a tightness in my chest that I had never felt before”* ^5^ *“I had to go to work, and I was forced to leave her alone at home. These thoughts make me agitated, anxious, stressed because even when I was at work I was always thinking about home”* ^6^ *“Then I felt helpless in the face of this thing, even for everyone else”*
Physical disconfort	
Sleep problems ^1^	Sleep problems
Feelings of tiredness	Feelings of tiredness ^3^
Psychosomatic reactions ^2^	
^1^ “*For a while I struggled to sleep and I had bad dreams… I slept badly at night”* ^2^ *“So it was a period when I really had this difficulty in moving, just a strong rigidity*”	^3^ “*The physical, moral and spiritual strengths were a bit lower… I felt tired and a bit frantic”*
Cognitive impact	
Concentration difficulties ^1^	Concentration difficulties ^3^
Attention problems	Attention problems
Feelings of confusion ^2^	Overthinking tendency ^4^
^1^ *“I was really stuck in everything, at work I had a lot of difficulties in understanding what I was doing, and even with the children, I had difficulties following the games we were playing at”* ^2^ *“Sometimes I felt a little confused due to the stress; on a cognitive level every now and then I lost a little*”	^3^ “*The drop in concentration because obviously my mind was racing to something else and even at work sometimes I lose track”.* ^4^ *“Having many thoughts, always mulling over the same thing”*
Coping strategies: Self-regulation	
Rationalization ^1^	Rationalization ^4^
Practicing hobbies and sports ^2^	Practicing hobbies and sports ^5^
Seeking information ^3^	Self-isolation ^6^
^1^ *“Keeping a little calm, rationalizing… I try to rationalize as much as possible without getting anxious”* ^2^ *“I started doing some sport, I went for a walk so I relaxed and it all went away”* ^2^ *“The only thing that really makes me switch off, that doesn’t make me think about the problem, is a good read. I managed to find moments in which I truly zoned out and immersed myself in reading”* ^3^ *“In order not to feel excessive anguish, I read news a lot, tried to understand the situation”*	^4^ *“I tried to react as coolly as possible, as if it hadn’t happened to me, so I was able to stay a little less emotionally involved”* ^5^ *“I tried to engage in other activities that weren’t just those related to taking care of my children and the house. I tried to go out every evening, walking, that was my lifeline!”* ^6^ *“I often also try to isolate myself a little to recharge and therefore to stay a little aside, in silence, I try to stay a little apart for those ten minutes,”*
Coping strategies: Other-regulation	
Searching for other people’s support	Searching for other people’s support
*“I asked for a laugh from my husband, from my daughter, support from my father… for better or worse, yes I had it, no one denied it”*	*“Oh well asked for help, and after that my husband and I started doing things inside the house”*
Parenting strategies	
Engaging in an open and transparent dialogue ^1^	Engaging in an open and transparent dialogue ^6^
Appeasing and reassuring ^2^	Appeasing and reassuring ^7^
Engaging in daily activities ^3^	Engaging in daily activities ^8^
Being tolerant and indulgent ^4^	Being tolerant and indulgent ^9^
Strengthening connection with the family ^5^	Strengthening connection with the family ^10^
^1^ *“I was at home a lot, supporting him and helping him. I tried to make him understand the situation, to explain to him what we were experiencing”* ^2^ *“I tried to reassure them by saying that it was a temporary thing, that everything passes… at least at school you can see your friends, you can talk to them, I tried to play it down a bit”* ^3^ *“This pressing request of mine for her to do things. I stimulated her to do more, in any case to commit herself more”* ^4^ *“He often wants to play with the tablet, if before I was more strict I made him do it more than necessary”* ^5^ *“We bonded a little more, before I was almost never at home, now the bond has become closer, we are here”*	^6^ *“I tried to impose my thoughts on her several times, other times I tried to get her to arrive at what I thought was the correct thing with reasoning, I realize that when I am calm we can reason and reach the goal together”* ^7^ *“I tried to make them understand that in any case it is not just their situation but it is a situation for everyone”* ^8^ *“I didn’t leave her alone, I tried to keep her active from all points of view, including mental ones, encouraging her to do things she doesn’t usually do, I kept her very busy”* ^9^ *“Once he has finished his school duties, I let him do it, he puts on his headphones and chats even until 10 pm”* ^10^ *“I try to encourage her to go out with her friends there when possible, even just for a walk so that she avoids social isolation.”*

Note. Same superscripts indicate correspondence between theme and quotes.

**Table 6:** Themes and Sub-themes with Quotes from Children’s and Adolescents’ Interviews.

Children	Adolescents
Emotional reactions	
Fear of contagion ^1^	Fear of contagion ^5^
Worries and anxiety ^2^	Worries and anxiety ^6^
Sadness and depressive mood ^3^	Sadness and depressive mood ^7^
Feeling of boredom and lack of fun moments ^4^	Feeling of boredom and lack of fun moments ^8^
^1^ *“I’m afraid of getting sick or that my family gets sick…the contagions were were growing in very high numbers so I was worried we could get it…then I play with my sister and all the worries go away.”* ^2^ *“I was concerned yes because there is always this thought: like if we don’t put on the mask or if we don’t wash our hands often, we can catch it. Luckly nothing has happened for now… but I’ve this thought of, of using the hand gel often, of washing often my hands, and at school I never put the mask down in fact I wear two.”* ^3^ *“I regret I can’t always hug my classmates, I regret I can’t do everything that used to be possible”* ^4^ *“I can’t go out…staying always inside my house the things I do are repetivive, I mean I always do the same things so after a while I get bored”*	^5^ *“I have so much fear of this virus, [I fear] to put my parents and my grandparents at risk”* ^6^ *“I’m so worried, I don’t feel like doing things and I spend much more time on my phone, given that we can’t go out and we can’t do anything, I waste my days doing almost nothing”* ^7^ *“Above all, I’ve felt alone because eemh before I could talk with my classmates about what happened to me, but during the lockdown I couldn’t talk.”* ^8^ *“I’ve had moments of dejection like when they closed my dance school. I feel empty when I dance at home cause..I get depressed, I’ve no idea of how to do it cause there’s not the same space nor the same interaction that there would be at the school, so I get depressed”*
Coping strategies: self-regulation	
Hobbies and diversions ^1^	Hobbies and diversions
Avoiding thinking about the situation	Keeping thoughts and feelings for themselves ^3^
Positive thinking ^2^	
^1^ “*I try to create diversions and not to think about these things, I get a little distracted, I try to distract myself*” ^2^ “*I think of the happy times when we used to go out without masks*”	^3^ “*I’m used to keep everything inside cause I dont’ feel the need to talk naaa I don’t feel like*”
Coping strategies: Other-regulation	
Activities with family members ^1^	Interaction with peers ^4^
Asking for help to family members ^2^	Asking for help to family members ^5^
Interactions with pets ^3^	Interactions with pets ^6^
^1^ “*I used to play with my sister, a little bit with mom, like I may have told her “Mom come on, let’s do something nice, let’s make a cake together or put a song on TV, let’s dance together”. Yeah if I’m really really sad 3I ask for her help*” ^2^ “*It has been of great support during this lockdown, I mean I was spending all the time with her, then in the evening I really vent [my emotions]*” ^3^ “*I took better care of my little dog*”	^4^ “*I kept in touch with friends through videocalls, calls o texts, sometimes even for some walls*.” ^5^ “*Sometimes my mum we do something togheter when I feel more stressed*” ^6^ “*I keep my cat close cause it helps to calm my stress*”

Note. Same superscripts indicate correspondence between theme and quotes.

## Data Availability

The data presented in this study are available on request from the corresponding author.

## Appendix A. Child and Adolescent’s Interview Protocol

**Table A1:** Child and adolescent’s interview protocol questions.

COVID-19 Difficulty Management
Have there been any difficulties, big or small, in your life since COVID began? Were there any difficulties in school? Were there difficulties at home? Were there difficulties with friends? What did you do to manage these situations?

**Table A2:** Emotional Reactions and Coping Strategies.

	Emotional Reactions and Coping Strategies
Stress	Since this COVID situation began, have you been feeling particularly stressed? What do you notice you are stressed by? What do you do to feel better?
Boredom	Since this COVID situation began, do you feel particularly bored? What do you notice you are bored by? What do you do to feel better?
Sadness	Since this COVID situation began, do you feel particularly sad? What do you notice you are sad by? What do you do to feel better?
Concern	Since this COVID situation began, do you feel particularly concerned? What do you notice you are concerned by? What do you do to feel better?

## Appendix B. Mother’s Interview Protocol

**Table A3:** Mothers’ Interview Protocol Questions: Emotional Reactions and Coping Strategies of Mothers Related to COVID-19 Difficulties.

Mothers’ Emotional Reactions And Coping Strategies
Over the past few months, have you been in difficulties with the COVID-19 pandemic? How did you react to these difficulties? How did you feel on a psychological level? How did you feel on a physical level? How did you feel on a cognitive level? What did you do to overcome that moment?

**Table A4:** Mothers’ Protocol Interview Questions: Children’s Difficulties Related to COVID-19 Recognized by Mothers and Parenting Strategies Used to Manage Them.

Recognition and Management of Children’s Difficulties		
Children’s difficulties	Social contest	Have you noticed difficulties in relationships with his/her friends? How did he/she react to them? How did he/she handle them?
	School contest	Have you noticed any difficulties in the school context? How did he/she react to them? How did he/she handle them?
	Family contest	Have you noticed any difficulties in the family context? How did he/she react to them? How did he/she handle them?
Parenting		With respect to the difficulties encountered by your children, you in general how did you deal with them?
